# New TB treatments hiding in plain sight

**DOI:** 10.15252/emmm.201404815

**Published:** 2014-12-22

**Authors:** Andrew J Olive, Christopher M Sassetti

**Affiliations:** 1 Department of Microbiology and Physiological Systems, University of Massachusetts Medical SchoolWorcester, MA, USA; 2 Howard Hughes Medical InstituteChevy Chase, MA, USA

## Abstract

As tuberculosis (TB) toll is revised upward according to the WHO's last estimates, the lack of vaccine strategy and the lengthy antibiotic treatments that unfortunately promote the emergence of drug resistance are a major set back in the fight against this pathogen. In this issue of *EMBO Molecular Medicine*, Schiebler *et al* (Mtb) propose a novel and compelling new approach to target *Mycobacterium tuberculosis* (Mtb) by pharmacologically stimulating intracellular mycobacteria clearance through autophagy.

See also: M Schiebler *et al* (February 2015)

The fitness of an intracellular bacterial pathogen is inextricably linked to the physiology of its host cell. Essentially, every aspect of the bacterium's environment is dictated by the metabolic state of this cell, which in turn is determined by a complex network of innate immune signaling pathways, external metabolic cues, and direct manipulation by the pathogen. This web of interconnected pathways is akin to the six degrees of separation that are said to separate any two individuals; systems that may seem unrelated share indirect, but functionally important, connections. Recent studies have elucidated unexpected connections between metabolism and immune defense and suggest that new treatments for tuberculosis may lay hidden in our existing pharmacopeia.

Infections with Mtb continue to be a major public health concern causing millions of deaths each year (2010). Factors limiting tuberculosis control include a huge burden of asymptomatic infection, the lack of a vaccine that protects from pulmonary disease, and lengthy antibiotic regimens that are being compromised by the continual emergence of drug-resistant strains (Casenghi *et al*, 2007; Nunes-Alves *et al*, 2014). Despite the tremendous burden of TB disease, most individuals infected with Mtb mount an effective immune response and remain asymptomatic for years or even a lifetime. Autophagy has emerged as a critical aspect of the protective host immune response (Bradfute *et al*, 2013). Mtb infection activates selective autophagy in phagocytes, also known as xenophagy, which has been proposed to serve an antimicrobial function that restricts bacterial replication by targeting the intracellular bacterium to the inhospitable autophagosome. Fundamental cell biological processes such as autophagy are modulated by many distinct inputs such as host metabolism, amino acid availability, oxygen levels, and innate immune pathways (Choi *et al*, 2013). This complex interplay between fundamental cell physiology and immune defense suggests that it may be possible to enhance immunity by indirectly manipulating cell metabolism.

In this issue of *EMBO Molecular Medicine*, Schiebler *et al* (2015) show that this is indeed possible. The authors screened a panel of FDA-approved drugs with no direct anti-bacterial activity for the ability to restrict the intracellular growth of mycobacteria. Two anticonvulsants, carbamazepine (CBZ) and valproic acid, were found to potently inhibit the replication of pathogenic mycobacteria in macrophages. Further characterization of CBZ function showed that this compound was able to restrict the growth of several distinct species of Mycobacteria in different eukaryotic hosts, including zebrafish and *Dictyostelium*, suggesting that an evolutionarily conserved pathway was being perturbed. CBZ treatment was even able to restrict the growth of antibiotic-resistant Mtb in mice suggesting an immediate therapeutic application (Schiebler *et al*, 2015).

CBZ is a sodium channel inhibitor that is commonly used to treat epilepsy and neuropathic pain. How many degrees of separation lie between these neurological effects and those that limit the replication of Mtb? When the authors examined the mechanism underlying CBZ activity, they uncovered a series of surprising connections. They found that in phagocytes, CBZ inhibits the sodium-dependent inositol transporter SCN5A. Blockade of this transporter decreased the intracellular pools of inositol leading to a concomitant decrease in PIP_2_ and IP_3_ that reduces mitochondrial Ca^2+^ and ATP levels. The drop in intracellular ATP subsequently activates AMP kinase (AMPK) and ULK1 to drive autophagy activation and mycobacterial killing (Fig1). This pathway is distinct from autophagy that is triggered by the classical activator, rapamycin, as CBZ treatment does not alter S6 and p70S6 kinase phosphorylation and is independent of mTOR.

**Figure 1:**
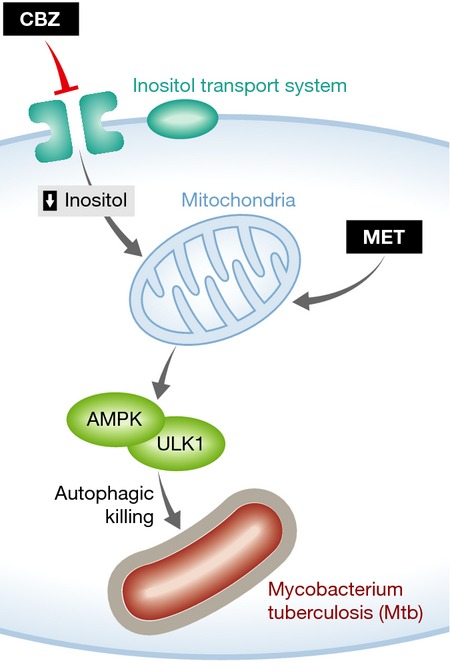
Metabolic modulators enhance selective autophagy Both CBZ and MET activate AMPK by perturbing mitochondrial function. The result of these effects is an enhancement of selective autophagy, which can both kill intracellular bacteria and alter cytokine secretion from phagocytes.

These elegant cell biological studies will serve as a starting point to understand the effects of CBZ *in vivo*. While the selective autophagy observed in cultured macrophages can serve a direct antimicrobial role, the effects of this ubiquitous process on the immune response are more complex. For example, much of the same cellular machinery that is involved in the clearance of intracellular bacteria also limits inflammation by disposing of damaged mitochondria (Choi *et al*, 2013). This functional overlap makes it difficult to ascribe the protective effects of autophagy *in vivo* to a simple antimicrobial activity. Indeed, previous studies suggest that regulation of the inflammatory response by autophagy may play a dominant role in protecting an intact animal from Mtb infection (Castillo *et al*, 2012). Similarly, Schiebler and colleagues find that treatment of infected macrophages with CBZ both increases Mtb killing and alters cytokine secretion (Schiebler *et al*, 2015). Understanding how long-term CBZ treatment influences immunopathology and tissue repair mechanisms is an important next step in understanding the benefit of this treatment.

Ultimately, any new treatment for TB would be used in the real world where genetic variation in host and pathogen, co-infections, and metabolic disorders all influence the expression of TB disease and could similarly influence the efficacy of a host-directed therapy. This is particularly relevant for CBZ, as several of these predisposing factors could directly influence autophagy. For example, the gene encoding the critical ubiquitin ligase, PARKIN, is functionally polymorphic in humans (Manzanillo *et al*, 2013). Similarly, the induction of STING-dependent autophagy relies on activity of the specialized type VII secretion system of the bacterium, which varies in different clinical isolates of Mtb (Watson *et al*, 2012). Finally, and perhaps most significantly, metabolic disorders such as diabetes mellitus are becoming the most significant risk factor for TB worldwide and are known to alter the same AMPK-dependent pathways that are ultimately impacted by CBZ (Martinez & Kornfeld, 2014). Thus, it seems likely that this type of host-directed therapy will be most useful when targeted to a specific population.

The critical interplay between metabolic and antimicrobial pathways in the phagocyte is highlighted by a second recent article, which describes the repurposing of another commonly used drug as an autophagy-inducing TB therapy (Singhal *et al*, 2014). Like CBZ, the anti-diabetic, metformin (MET), has the ability to induce autophagy in an mTOR-independent manner by activating AMPK (Fig1). Singhal *et al* report that this compound also shares the ability to restrict Mtb growth in both macrophages and mice. Importantly, metformin-containing regimens improve TB outcome in diabetic patients, validating AMPK activators such as MET and CBZ as candidate adjunctive TB therapies, particularly in the context of diabetes.

The remarkable protective effect of CBZ and MET during Mtb infection was unanticipated. However, given the central role of autophagy in both maintaining phagocyte physiology and promoting antimicrobial activity, the functional connections between metabolism and immunity that are described in these studies should not have been surprising. Indeed, as the intricate connections between metabolism, cell biology, and immunity become more apparent, the distinctions between antibiotics and modulators of host cell function may become increasingly blurred.
